# H. L. Evans

**Published:** 1925

**Authors:** 


### H. L. EVANS, M.B., C.M. Ed.

THE death of Herbert Lavington Evans, of Goring-on-Thames, on April 9th, at the age of 65, due to acute pneumonia following influenza, is a grave sorrow to his old Bristol Medical School friends. He was educated at Clifton College, UniversityCollege, Bristol, Edinburgh University and at Guy's Hospital. He was a Clinical Assistant at the Bristol Eye Hospital before he settled in practice at Goring-on-Thames thirty-six years ago, and where he was beloved and held in the highest esteem by a very large number of patients. In his younger days he was a fine athlete and distinguished himself at football firstly at Clifton College and later he played for Scotland in International Rugby football matches for three seasons. H. L. Evans was a man of great charm of character and ever a friend to depend on.

